# A urethral leiomyoma presenting with dysuria: A rare case report

**DOI:** 10.1097/MD.0000000000037893

**Published:** 2024-05-17

**Authors:** Shuo Wu, Zhichao Min, Lingyan Wu, Mengsi Zhang, Lejun Wu

**Affiliations:** a Department of Urology, The First Peoples’ Hospital of Hangzhou Linan District, Hangzhou, China; b Department of Ultrasound, The First Peoples’ Hospital of Hangzhou Linan District, Hangzhou, China; c Department of Pathology, The First Peoples’ Hospital of Hangzhou Linan District, Hangzhou, China.

**Keywords:** 3D pelvic ultrasound, acute urinary retention, dysuria, surgical treatment, urethral leiomyoma, urethral mass

## Abstract

**Rationale:**

Leiomyoma is a benign smooth muscle tumor which is rarely found in urethra. We hereby report a case of a 44-year-old female who presented with complaints of dysuria.

**Patient concerns:**

A 44-year-old female patient presented to the urology outpatient clinic with symptoms of dysuria. The patient described the presence of a protrusion from the urethra during urination.

**Diagnosis:**

Urethral leiomyoma.

**Interventions:**

Physical examination confirmed a solid urethral mass. CT scan and USG reports indicated that the mass originated from the mid-urethra with vascularity at the base. We performed a complete resection of the urethral mass. The patient was discharged after 3 days of observation.

**Outcome:**

During a follow-up after 1 month, the patient reported improved urinary flow and no occurrence of hematuria. The patient recovered well after discharge.

**Lesson:**

Urethral leiomyoma is a rare benign tumor that is often misdiagnosed in clinical practice. Diagnosis requires careful clinical examination. Surgical removal usually works well. It is important to remember that in some cases of acute urinary retention, it can be caused by a complete obstruction of a mass in the urethra. Urologists should be more cautious and experienced in handling such cases.

## 1. Introduction

Urethral leiomyoma is an unusual leiomyoma type, with fewer than 100 cases reported to date. These benign mesenchymal leiomyomas arise from the smooth muscle of the female urethra.^[1,2]^ Urethral leiomyomas usually manifest as a protruding mass from the urethral meatus, accompanied by symptoms such as urethral bleeding, dysuria, and dyspareunia. Obstructive voiding,^[3]^ although rare, may also be present.

## 2. Case report

**Figure 1.**
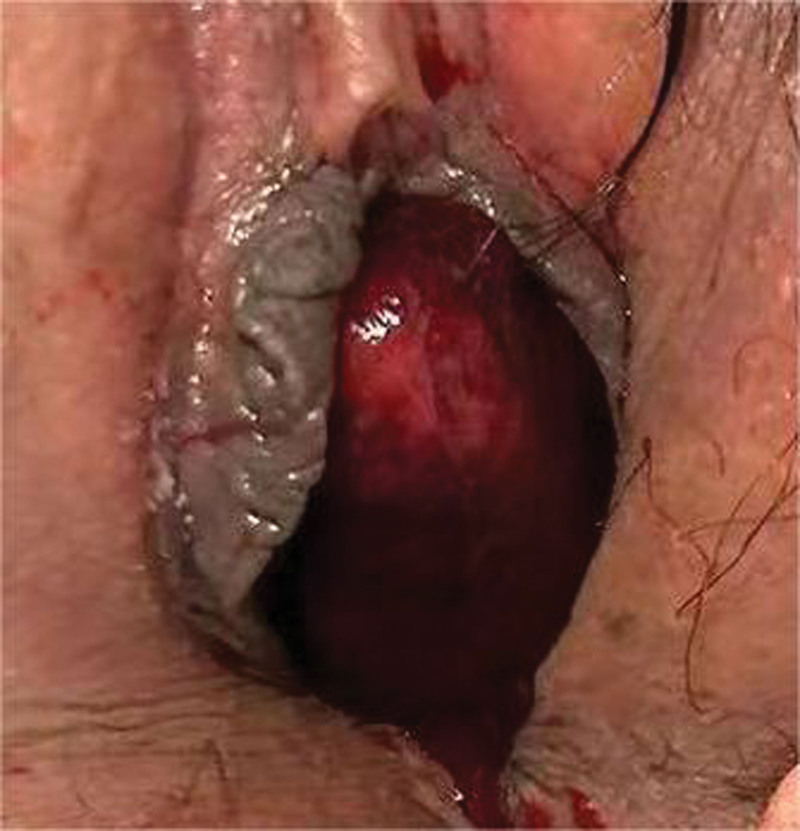
Urethral meatus rubbery and crineous mass with size of 2.0 cm × 2.0 × 2.5 cm.

**Figure 2.**
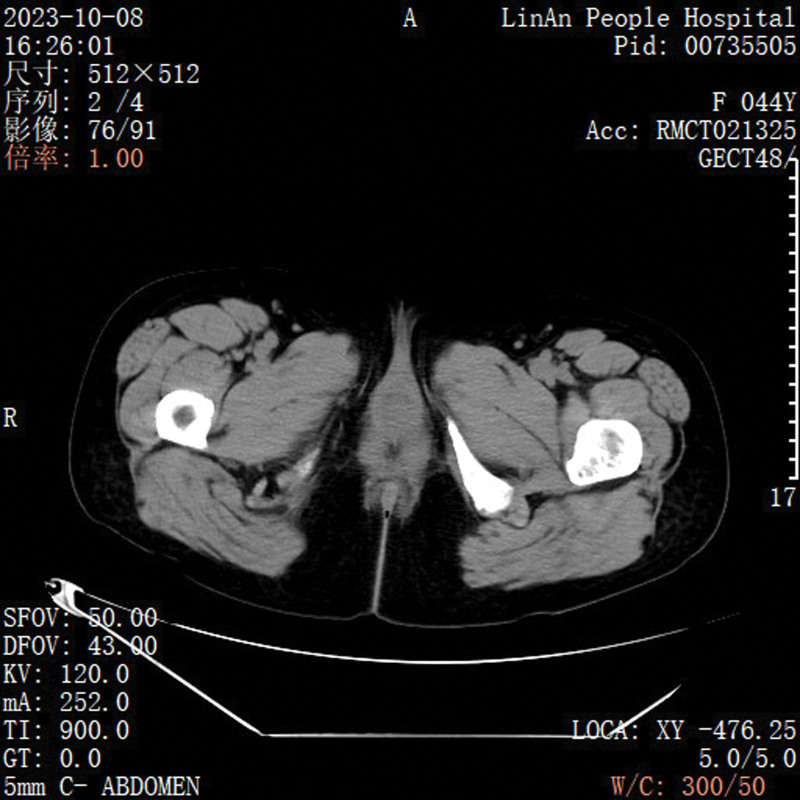
CT scan revealed that the tumor originates from the urethra. CT = computed tomography.

**Figure 3.**
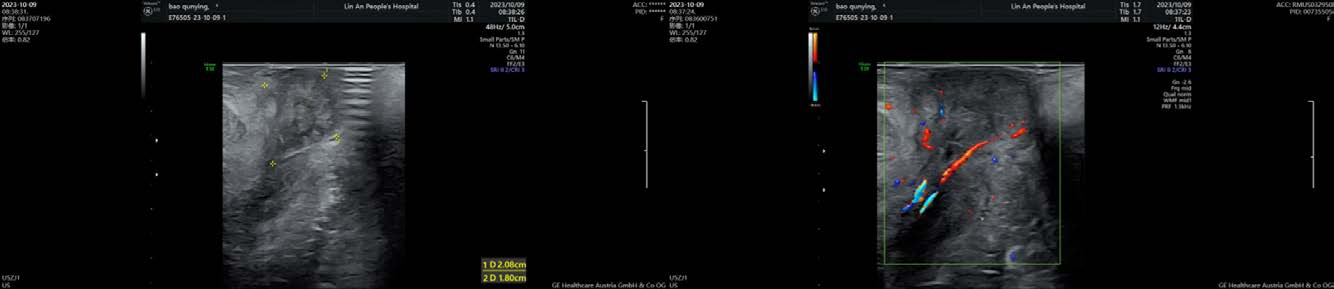
3D pelvic ultrasound examination indicated that the tumor originated from the mid-urethra with vascularity at the base.

A 44-year-old female patient presented to the urology outpatient clinic with symptoms of urinary difficulty. The patient has signed an informed consent and this study was approved by the Ethics Committee of the First People Hospital of Lin’an District. The patient described the presence of a protrusion from the urethra during urination (Fig. 1), accompanied by bloodstains and mild pain upon wiping. The patient reported a similar episode of symptoms 10 days ago, which resolved as the protrusion retracted on its own. However, this time the protrusion did not retract, leading the patient to seek medical attention. The patient denied any history of chronic disease. The outpatient examination confirmed that the patient vital signs, such as blood pressure and heart rate, were within normal range. A joint physical examination by the urologist and gynecologist revealed a solid, pedunculated, dark red mass measuring approximately 2.0*2.0*2.5 cm at the external urethral orifice. Subsequently, a vaginal examination using a speculum revealed no abnormalities within the vagina. The patient was admitted to the hospital for further evaluation, including pelvic ultrasound and CT scans (Figs. 2 and 3). The reports indicated that the mass originated from the mid-urethra with vascularity at the base. However, all imaging studies did not reveal any abnormalities in other pelvic organs, including the uterus, bladder, and vagina.

**Figure 4.**
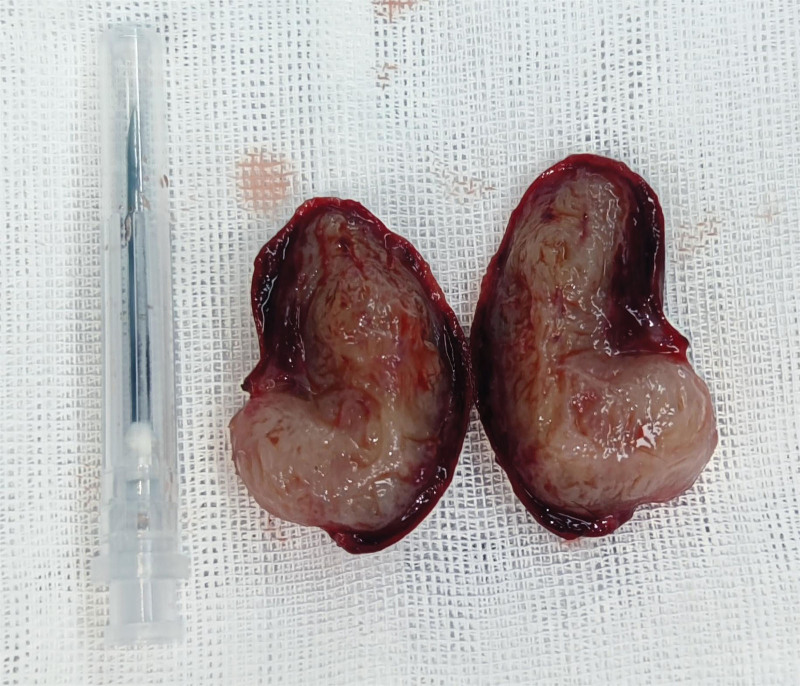
Surgical removal of tumor with a fish-flesh cut surface.

**Figure 5.**
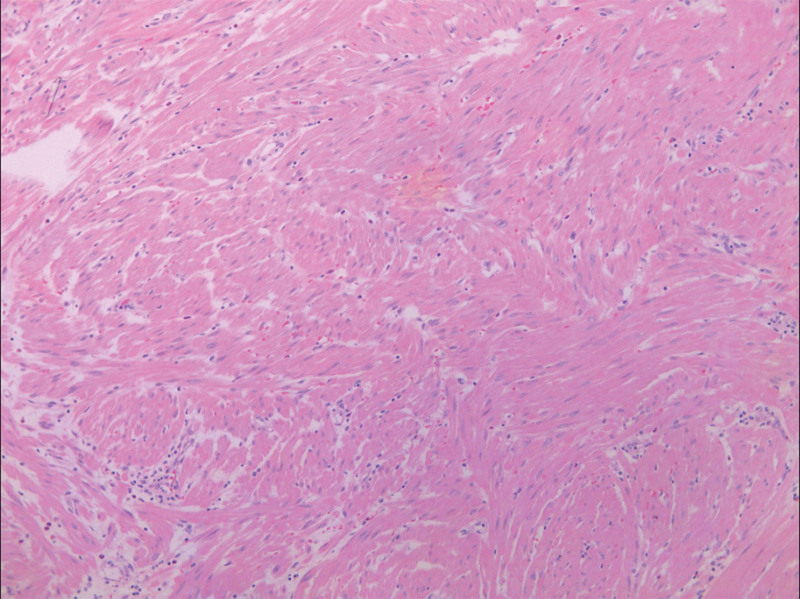
The tumor is composed of spindle-shaped cells arranged in intersecting bundles (40×).

Under spinal anesthesia, complete removal of the urethral mass, including the mass and its base tissue, was performed (Fig. 4). The resected specimen was sent for pathological examination. Post-surgery, cystoscopy was performed to confirm the integrity of the urethra and bladder. An F16 Foley catheter was subsequently placed and removed on the third postoperative day, allowing for a smooth discharge of the patient. The pathological report confirmed a urethral leiomyoma (Fig. 5).

After surgical catheter removal, the patient promptly exhibited improved urinary function. Early postoperative dysuria was brief and resolved rapidly. The patient reported a substantial increase urine flow rate and a significant enhancement in urination satisfaction. Following surgery, the patient received only antibiotic therapy. The urethral incision demonstrated excellent healing. At the 3-month follow-up, the patient maintained optimal urinary function with no signs of recurrence.

## 3. Discussion

Urethral leiomyoma is a rare benign mesenchymal leiomyoma that, like other leiomyomas, originates from tissues containing smooth muscle.^[4]^ Leiomyomas can be categorized into 3 types: cutaneous leiomyoma, vascular leiomyoma, and deep soft tissue leiomyoma.^[5]^ Urethral leiomyoma, in particular, belongs to the category of deep soft tissue leiomyoma. The most common type of leiomyoma in the human body is uterine leiomyoma, but it can also occur in uncommon sites such as the external genitalia, ovaries, urethra, or bladder.^[6]^ Urethral leiomyoma often occurs in female patients aged 20 to 50, while it is exceptionally rare in males. In this case, the patient is a female and falls into the high-risk age group.

Some studies propose a correlation between the development of urethral leiomyoma and estrogen levels in the body, same to uterine leiomyoma.^[7,8]^ Previous research has confirmed that a significant proportion of urethral leiomyoma samples express estrogen receptors (ER), indicating a potential role of the ER signaling pathway in leiomyoma progression. Furthermore, some patients experience rapid growth of urethral leiomyoma during pregnancy in some previous reports.^[9]^ This could be attributed to fluctuations in estrogen receptor levels in the urethral tissue, which like in the uterus and breast, vary with the menstrual cycle. During periods of high estrogen levels, the growth of urethral leiomyomas is stimulated. In this case, the patient was not pregnant, and there were no specific observations related to her menstrual cycle, but the pathological results indicated positive ER expression. However, the pathogenesis of urethral leiomyoma requires further in-depth research to clarify.

Urethral leiomyoma can occur in different locations within the urethra. In females, it is more commonly found in the anterior urethra, while in males, it is predominantly seen in the prostatic urethra and navicular fossa. The size of the leiomyoma is generally around 2 to 4 cm in diameter and rarely exceeds 5 cm. The symptoms experienced by patients and the size and location of the leiomyoma are closely related. Approximately 25% of urethral leiomyoma patients are asymptomatic, while others may present with symptoms such as urethral mass, hematuria, acute urinary retention, recurrent lower urinary tract infections, or vaginal bleeding.^[10,11]^ In this case, the patient presented with urinary difficulty, and the mass was located in the distal urethra, with the ability to self-retract. The onset of symptoms during this medical visit was sudden, but the condition remains relatively asymptomatic when there are no symptoms.

Although there is no specific consensus or guidelines, surgical treatment is generally the primary approach for urethral leiomyoma. The treatment modality depends on the location of the leiomyoma. Leiomyomas located near the internal urethral meatus can be treated using endoscopic resection, while those closer to the external urethral orifice require direct surgical excision. Some reports have described the use of laparoscopic surgery for larger urethral leiomyomas.^[12]^ In cases of leiomyoma infiltration or invasion discovered during the surgery, intraoperative rapid pathological examination can help confirm the nature of the leiomyoma, and if necessary, more extensive resection surgery may be performed. During routine surgery, attention should be paid to removing the base of the leiomyoma to prevent recurrence. Concerns about urethral damage during surgery can serve as evidence to distinguish urethral leiomyoma from periurethral leiomyoma.^[13]^

Differential diagnoses for urethral masses include urethral prolapse, urethral abscess, diverticulum, paraurethral cyst, polyp, papilloma, vascular malformation, leiomyoma, fibroma, neurofibroma, adenoma, and other leiomyomas. Malignant diagnoses may include leiomyosarcoma, lymphoma, transitional cell carcinoma, squamous cell carcinoma, clear cell adenocarcinoma, and metastasis.^[14]^ Postoperative pathological examination provides definitive evidence for diagnosing urethral leiomyoma, but preoperative evaluations can be improved by using pelvic ultrasound or magnetic resonance imaging to provide preliminary information about the leiomyoma. In this case, the patient did not undergo a magnetic resonance imaging examination due to personal reasons. However, a preoperative 3-dimensional pelvic ultrasound examination provided valuable information. It revealed that the leiomyoma had a pedicle and originated from the middle segment of the urethra and the vaginal hiatus. The presence of blood flow signals within the base and clear delineation between the urethral leiomyoma and surrounding tissues were observed. There was no infiltration or adhesion, which carried significant importance for preoperative leiomyoma assessment. At the 3-month follow-up, there was no leiomyoma recurrence, but long-term efficacy requires further observation.

This study also has some limitations, such as a small, singular sample and the absence of a comparison group. The therapeutic approach, derived from clinical practice, may influence the prognostic outcomes. The descriptive nature of case reports, without randomization or blinding, could introduce bias. Moreover, the brief follow-up duration limits insights into sustained efficacy and potential recurrence.

## 4. Conclusion

Urethral leiomyoma is a rare benign tumor that often misdiagnosed in clinical practice, before pathological reports are available. Diagnosis requires careful clinical examination. Surgical resection typically results in good outcomes. It is important to keep in mind that in some cases of acute urinary retention, it may be caused by complete obstruction from urethral mass. Urologists should be more careful and experienced in managing such cases.

## Acknowledgments

The authors appreciate the patient consent to present this case.

## Author contributions

**Conceptualization:** Shuo Wu, Lejun Wu.

**Data curation:** Lingyan Wu, Mengsi Zhang.

**Formal analysis:** Lingyan Wu, Mengsi Zhang.

**Investigation:** Zhichao Min.

**Visualization:** Lingyan Wu, Mengsi Zhang.

**Writing – review & editing:** Shuo Wu, Zhichao Min, Lejun Wu.
